# Betel nut chewing, oral premalignant lesions, and the oral microbiome

**DOI:** 10.1371/journal.pone.0172196

**Published:** 2017-02-22

**Authors:** Brenda Y. Hernandez, Xuemei Zhu, Marc T. Goodman, Robert Gatewood, Paul Mendiola, Katrina Quinata, Yvette C. Paulino

**Affiliations:** 1 University of Hawaii Cancer Center, Honolulu, Hawaii, United States of America; 2 Cedars-Sinai Medical Center, Los Angeles, California, United States of America; 3 University of Guam Cancer Research Center, Mangilao, Guam, United States of America; University of Illinois at Urbana-Champaign, UNITED STATES

## Abstract

Oral cancers are attributed to a number of causal agents including tobacco, alcohol, human papillomavirus (HPV), and areca (betel) nut. Although betel nut chewing has been established as an independent cause of oral cancer, the mechanisms of carcinogenesis are poorly understood. An investigation was undertaken to evaluate the influence of betel nut chewing on the oral microbiome and oral premalignant lesions. Study participants were recruited from a dental clinic in Guam. Structured interviews and oral examinations were performed. Oral swabbing and saliva samples were evaluated by 454 pyrosequencing of the V3- V5 region of the 16S rRNA bacterial gene and genotyped for HPV. One hundred twenty-two adults were enrolled including 64 current betel nut chewers, 37 former chewers, and 21 with no history of betel nut use. Oral premalignant lesions, including leukoplakia and submucous fibrosis, were observed in 10 chewers. Within-sample bacterial diversity was significantly lower in long-term (≥10 years) chewers vs. never chewers and in current chewers with oral lesions vs. individuals without lesions. Between-sample bacterial diversity based on Unifrac distances significantly differed by chewing status and oral lesion status. Current chewers had significantly elevated levels of *Streptococcus infantis* and higher and lower levels of distinct taxa of the *Actinomyces* and *Streptococcus* genera. Long-term chewers had reduced levels of *Parascardovia* and *Streptococcus*. Chewers with oral lesions had significantly elevated levels of *Oribacterium*, *Actinomyces*, and *Streptococcus*, including *Streptococcus anginosus*. In multivariate analyses, controlling for smoking, oral HPV, *S*.*anginosus*, *and S*. *infantis* levels, current betel nut chewing remained the only predictor of oral premalignant lesions. Our study provides evidence that betel nut chewing alters the oral bacterial microbiome including that of chewers who develop oral premalignant lesions. Nonetheless, whether microbial changes are involved in betel nut-induced oral carcinogenesis is only speculative. Further research is needed to discern the clinical significance of an altered oral microbiome and the mechanisms of oral cancer development in betel nut chewers.

## Introduction

Oral cancers, comprising tumors of the oral cavity and oropharynx, are among the most common malignancies worldwide.[1] Oral cancers have a multifactorial etiology and risk factors vary across different parts of the world. Historically, tobacco and alcohol use have been primary causes of oral cancers across the globe.[2] Human papillomavirus (HPV), primarily genotypes 16 and 18, has been increasingly recognized as a causal agent in oropharyngeal and, to a lesser extent, oral cavity tumors, particularly in North America. [3, 4]

Areca (betel) nut chewing is a leading cause of oral cancer in parts of Asia and the Pacific. [2] Chewing of betel nut, which comes from the *Areca catechu* palm tree, is practiced by 10%-20% of the world’s population with the highest prevalence of use in South and Southeastern Asia and the Pacific.[2, 5] Worldwide, the highest incidence of oral cavity tumors is found in Melanesia, including Papua New Guinea and the Solomon Islands, where betel nut chewing is widely used.[1, 2, 6] In Guam, a U.S. territory in the western Pacific where betel nut chewing is prevalent [7], oral cancer mortality rates among the native Chamorro population are six times higher than that of the U.S.[8]

Betel nut chewing is considered the fourth most commonly used addictive substance in the world after tobacco, alcohol, and caffeine.[5] Chewing typically initiates in youth or early adulthood and progresses to habitual, regular betel nut use which continues over many years.[6] The major chemical components of betel nut are polyphenols, including tannins, and alkaloids. Arecoline, the primary alkaloid, is a muscarinic acetylcholine receptor agonist producing cholinergic effects on the parasympathetic nervous system and a psychoactive agent.[9]

The underlying mechanisms linking betel nut chewing to oral carcinogenesis are not well understood. The *Areca* nut can be chewed whole or cut in half or as “quid”, a mixture containing crushed *Areca* nut combined with betel leaf, tobacco, slaked lime, alcohol, or other substances.[6, 7] Betel nut chewing is causally linked to cancers of the oral cavity when used alone or mixed with tobacco and/or alcohol.[2] Regular chewing may induce chronic irritation and inflammation that damage epithelial cells of the oral cavity. [2] Arecoline and/or other areca nut components have been shown to induce a number of pro-carcinogenic changes including the production of nitrosamines and reactive oxygen species, modulation of matrix metalloproteinases and their tissue inhibitors, inhibition of collagenases and increased collagen cross-linkage, up-regulation of heat-shock proteins [2] and integrins [10], and increased expression of inflammatory cytokines, including tumor necrosis factor-α, interleukin-1-β, interleukin- 6, and interleukin-8. [11] Elevated sister-chromatid exchange and micronucleus formation have been demonstrated in cultured peripheral lymphocytes of chewers. [12]

The potential role of the oral bacterial microbiome in betel-nut related oral carcinogenesis is relatively unexplored. Over 300 bacteria inhabit the oral cavity of healthy individuals and most are commensals which play an important role in maintaining homeostasis including protection against pathogenic species, down-regulation of inflammation including proinflammatory cytokine production, and reduction of nitrate and nitrite to nitrogen oxide and other reactive nitrogen intermediates.[13–15] Betel nut chewers often experience poor oral hygiene [16] and chronic periodontitis [16, 17], both of which have been linked to changes in oral bacterial composition and oral cancer risk.[18, 19]

The early development of oral cancer in betel nut chewers typically manifest as specific lesions of the oral cavity including leukoplakia, erythroplakia, and oral submucous fibrosis, the precursor lesion most strongly linked to oral cancer in betel nut chewers.[2, 18, 20] Specific bacteria have been identified in the development of oral premalignant lesions including oral submucous fibrosis. [18] Pathogenic bacterial species are more prevalent in oral samples from patients with oral squamous carcinoma compared to healthy controls.[21–23] In oral cancer patients, differences have been observed in the microbial composition of tumor and precancerous tissue compared to non-tumor tissue.[24–28]

An investigation was undertaken in Guam to evaluate the influence of betel nut chewing on the oral microbiome and oral premalignant lesions in the context of tobacco and alcohol use, oral HPV infection, and other factors.

## Materials and methods

### Study population

The study was approved by the Institutional Review Boards of the University of the Hawaii Cancer Center (WIRB# 20121912) and the University of Guam (CHRS# 12–129). Written informed consent was obtained from all individuals. A convenience sample of 122 study subjects were recruited from patients seen in at dental clinic in Guam between July 2013 and October 2014. Clinic attendees at least 18 years of age and able to understand English were eligible to participate. The present report is limited to the baseline visit and does not include results from a subset of subjects followed at subsequent visits.

### Data and oral specimen collection

A structured interview (**S1**) was administered by research staff to collect information on demographics, medical history, height and weight, smoking and alcohol use, and betel nut use, including the duration and frequency of consumption and the added use of Piper betel leaf, slaked lime, and tobacco, and alcohol. The presence of oral lesions was evaluated and documented by a registered dental hygienist and confirmed by a periodontist, based on a screening protocol described in detail elsewhere.[29] Oral cell swabbings and saliva samples were collected by research staff using a protocol adapted from the NIH Human Microbiome Project[30] http://hmpdacc.org/doc/HMP_MOP_Version12_0_072910.pdf. Briefly, 3–5 ml saliva was collected into a sterile collection tube. A foam swab was then used to sample the center of the tongue, below the tongue, hard palate, buccal mucosa, and upper front gums and placed into a separate sterile collection tube. Samples were stored at minus 20 degrees Celsius until shipment on ice to the University of Hawaii Cancer Center for testing.

### 16S rRNA PCR and 454 pyrosequencing

DNA extraction, 16S rRNA PCR, and 454 pyrosequencing were based on protocols established by the NIH Human Microbiome Project (HMP). [30, 31] http://hmpdacc.org/doc/HMP_MOP_Version12_0_072910.pdf http://hmpdacc.org/doc/16S_Sequencing_SOP_4.2.2.pdf. Total DNA was extracted from oral cell and saliva samples using commercial reagents (PowerSoil DNA Isolation Kit, MoBio, Carlsbad, CA). Due to cost limitations, extracted DNA from saliva and oral swabs samples were combined for PCR and sequencing rather than separately assayed. Negative controls (sterile water) and positive controls (*Campylobacter coli* DNA; ATCC, Manassas, Virginia), selected based on the NIH HMP protocols, were included in PCR and sequencing assays. The PCR assay targeted the V3-V5 regions of the bacterial 16S ribosomal RNA (rRNA) gene. Individually barcoded universal primers 357F and 926 R (V3-V5) containing the A and B sequencing adaptors (Thermo Fisher Scientific /Life Technologies Corporation, Waltham, MA. USA) were used. Amplicons were cleaned (Agencourt AMPure, Beckman Coulter, Beverly, MA), quantified on a NanoDrop 2000 spectrophotometer, and diluted to obtain equimolar concentrations. Samples (200 ng each) were pooled followed by purification and concentration (MinElute, Qiagen, Inc., Valencia, CA., USA). Following pool emulsion PCR, 454 pyrosequencing were performed on a Roche 454 Life Sciences Genome Sequencer FLX instrument at the University of Hawaii Sequencing Core Facility. Samples were sequenced in 5 separate runs.

### HPV genotyping

Extracted DNA from oral samples were evaluated for HPV DNA using previously described methods.[32] Briefly, oral DNA was assayed by PCR targeting HPV L1 and amplicons were genotyped with the Linear Array HPV Genotyping Test (LA, Roche Diagnostics, Indianapolis, IN), which distinguishes 37 HPV genotypes. Human beta-globin PCR was included as a measure of sample sufficiency.

### Data processing and statistical analysis

Data was processed and analyzed using the Quantitative Insights into Microbial Ecology (QIIME, v.1.7.0.[33] software. SAS version 9.4 (SAS Institute, Carey, North Carolina) was also used for statistical analyses of processed data. Statistical significance of p< 0.05 was used for all analyses. Raw sequences were processed based on the standard QIIME workflow.[33, 34] Briefly, sequences were quality-filtered to remove sequences with less than 200 nucleotides, ambiguous bases, and sequences with a quality score of less than 25. Multiplex reads generated from the same samples were grouped by barcode; barcode adapter and reverse primer sequences were then removed. Filtered sequences were denoised using QIIME's built-in denoiser. Filtered sequences from the 5 runs were combined. Sequences were then clustered into Operational Taxonomic Units (OTUs) via QIIME’s de novo OTU picking strategy at the 97% similarity level; chimeras were removed using uclust.[35] Representative sequences for each OTU were selected and aligned using pyNAST [33] against the Greengenes database.[36] Taxonomic assignments of OTUs were made based on the representative sequence using the Ribosomal Database Project (RDP) Bayesian classifier.[37] Sequences were used to construct phylogenetic trees for calculation of UniFrac distances. Data was rarefied based on an average of 10 iterations in which 100 sequences were randomly sampled from each specimen and rarefied OTU tables were generated.

The adequacy of sampling was estimated using Good’s estimator of coverage.[38] Within-sample (alpha) diversity, a measure of sample richness (number of unique taxa) and evenness (relative abundance of unique taxa) was evaluated using Shannon’s Index,[39] an OTU-based measure of taxa richness and evenness, Chao1,[40] a measure of taxa richness, Observed Species, a count of unique OTUs in a sample; and Faith’s Phylogenetic Diversity (PD) Whole Tree index,[41, 42] a measure of the phylogenetic branch length.

Between-sample (beta) diversity, was evaluated based on UniFrac distances representing the fraction of the branch length of the phylogenetic tree that is shared between groups. Both unweighted UniFrac distances and UniFrac distances weighted by the relative abundance of taxa were calculated.[43, 44] Unweighted UniFrac distance is able to detect abundance changes in rare taxa while weighted UniFrac distance is more sensitive to changes in abundant taxa.[43, 44] Three-dimensional Principal Coordinate Analysis (PCoA) was used to generate UniFrac scatterplots to visually compare microbial composition across groups. Beta diversity was evaluated using non-parametric, permutation-based tests including Permutational Multivariate Analysis of Variance Using Distance Matrices (PERMANOVA),[45] Analysis of Similarity ANOSIM,[46] Multi-response Permutation Procedure (MRPP),[47] and Permutation Test for Homogeneity of Multivariate Dispersion (PERMDISP) [47]. PERMDISP tests for homogeneity of group variances. All tests were based on 999 permutations.

Specific bacterial taxa, represented by individual OTUs, were compared. The presence or absence of OTUs was compared across groups using the g test of independence. The relative abundance of OTUs was compared across groups using ANOVA. G test and ANOVA analyses accounted for multiple comparisons with control for false discovery rate (FDR).

The relationship of premalignant oral lesions with chewing status, specific bacterial taxa, and other factors was evaluated. Univariate and multivariate unconditional logistic regression modeling was used to yield odds ratios and 95% confidence intervals.

## Results

### Characteristics of study population

A total of 122 adults were enrolled including 66 males and 56 females (**Table 1**). Nearly all study subjects were of Pacific Island ancestry, including Chamorros (60%), the major ethnic group indigenous to Guam. Sixty-four individuals reported current use of betel nut, 37 were former chewers, and 21 had no history of betel nut use. Of the 64 current chewers, 39 had used betel nut for 10 or more years and 40 chewed on a daily basis. Among current chewers, betel quid was used in combination with slaked lime (50%), tobacco (48%), and Piper betel leaf (56%). Only 1 chewer used alcohol in the betel quid. Of the 122 subjects, 29% reported current cigarette smoking, 57% reported current alcohol consumption, and 46% were obese. Antibiotics use within the prior 6 months was reported by 14% of participants. Betel nut use did not significantly differ by age, gender, antibiotic use, alcohol consumption, cigarette smoking, diabetes history, or oral HPV DNA status (data not shown). Differences were observed by body mass index (BMI): Of current betel nut chewers, 92% (58/62) were overweight or obese (BMI > 25 kg/m^2^) compared to 76% (28/37) of past chewers and 62% (13/21) of never chewers) (p = 0.003).

**Table 1:** Characteristics of Study Subjects (n = 122).

	No.	%
**Age**		
18–29	37	30.3
30–39	26	21.3
40–49	20	16.4
50–59	20	16.4
60+	19	15.6
**Sex**		
Male	66	54.1
Female	56	45.9
**Ethnicity**		
Chamorro	73	59.8
Other Pacific Islander	30	24.6
Asian	13	10.7
Caucasian	6	4.9
**Betel nut chewing**		
Ever	101	82.8
Current	64	52.5
Past	37	30.3
Never	21	17.2
**Duration of betel nut chewing** (current chewers)		
<10 years	25	39.1
≥10 years	39	60.9
**Frequency of betel nut use** (current chewers)		
Daily	40	62.5
Weekly	12	18.8
Monthly	12	18.8
**Substances added to betel nut** (current chewers)		
Slaked lime	32	50.0
Betel leaf	36	56.2
Tobacco	31	48.4
**Current cigarette smoking**	35	28.7
**Current alcohol consumption**	69	56.6
**Antibiotics within past 6 months**	17	13.9
**Type 2 diabetes**	10	8.2
**Body mass index (kg/m**^**2**^**)**^**1**^		
<18.5	1	0.8
18.5–24.9	21	17.4
25.0–29.0	43	35.5
≥30.0	56	46.3

### Oral premalignant lesions

**Fig 1:**
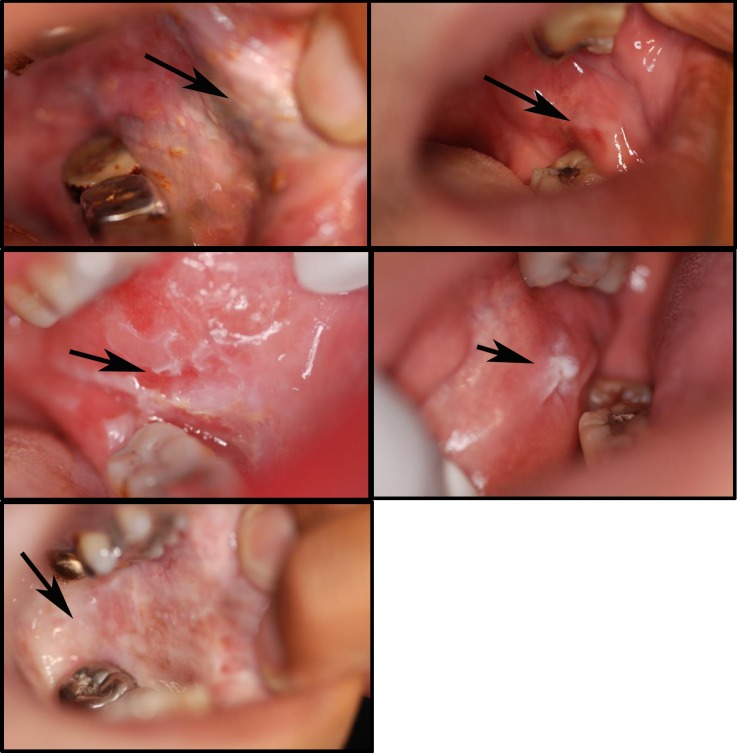
Oral lesions in betel nut chewers (left to right, beginning with top left): **a.** Striated leukoplakia with small ulcerations on buccal mucosae; **b.** Red erythroplakia of buccal mucosae; **c.** Mixed erythroplakia of buccal mucosae; **d.** Pedunculated leukoplakia of buccal mucosae; **e.** Submucous fibrosis with leukoplakia of buccal mucosae

No individuals had a history of oral cancer. Oral premalignant lesions were observed in 10 individuals including 9 current chewers and 1 past chewer; 7 had chewed for 10 years or longer. Oral lesions included leukoplakia, erythroplakia, and submucous fibrosis (**Fig 1A–1E**). The presence of oral premalignant lesions significantly varied by chewing status: lesions were present in 9 of 65 (14%) of current chewers, 1 of 37 (3% of past chewers), and none of the never chewers (p = 0.0432).

### Oral HPV DNA

HPV DNA was detected in 14% (17/122) of oral samples. HPV genotypes included oncogenic types HPV 66 and 68, and HPV 62, 82, 83, and 84, which are non-oncogenic.[48] Ten HPV DNA-positive samples were not positive for any of the 37 genotypes detected by the assay. The presence of oral HPV did not vary by betel nut chewing history.

### Oral bacterial taxonomy

A total of 1,036,083 raw sequences were generated from oral specimens of the 122 study subjects. After quality filtering, a total of 560,475 sequences remained for analyses. Each individual yielded an average of 3,591 (std. dev. 1,842) quality-filtered sequences with a mean sequence length of 452 bp. Four samples yielded ≤100 sequences and were excluded from diversity and taxonomic comparisons. The complete sequencing dataset was submitted to the National Center for Biotechnology Information (NCBI) (Biosample accession number SAMN06127413).

A total of 9,832 unique OTUs were generated with an average of 68 (std 2.2) OTUs per person. Good’s coverage averaged 90% across specimens. Overall, 100% of OTUs were classified at the phylum level; 80% were classified at the genus level; and only 11% were classified at the species level. A total of 12 phyla, 99 genera and 79 species were identified in oral samples. Taxonomic levels below phylum and above genus, e.g., class, order, and family, were not evaluated.

**Fig 2:**
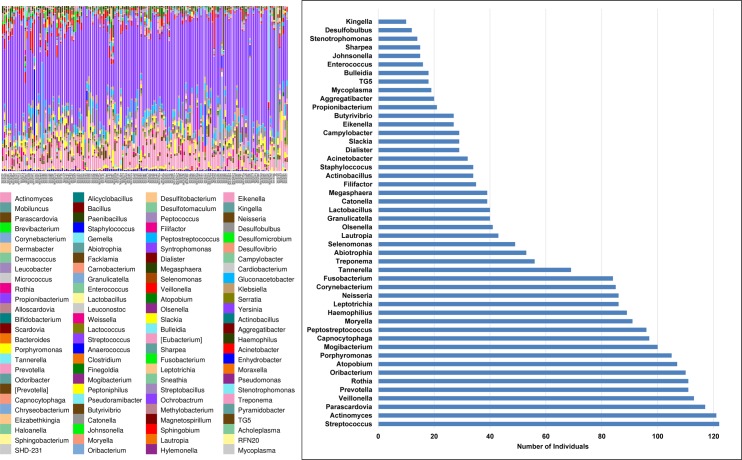
**a.** Genus-level oral microbiome composition: 99 genera including Streptococcus (53%), *Actinomyces* (8%), *Prevotella* (4%), and *Parascardovia* (2%); **b.** The most abundant genera were detected in the majority of individuals (excluding genera detected in fewer than 10 individuals).

Of the 12 phyla, *Firmicutes* was the most predominant comprising 75% of sequences followed by *Actinobacteria* (13%) *Bacteroidetes* (7%), and *Proteobacteria* (3%). *Firmicutes* and *Actinobacteria* were detected in all subjects and *Bacteroides* in all but 1 individual. Streptococcus was the most abundant genus comprising 53% of taxa followed by *Actinomyces* (8%), *Prevotella* (4%), and *Parascardovia* (2%), all of which were detected in nearly all subjects. (**Fig 2A and 2B**). At the species level, the highest relative abundances were observed for *Prevotella melaninogenica (3%)*, *Streptococcus infantis (3%)*, and *Veillonella dispar (1%)*. Seven *streptococcal* species were identified including *Streptococcus infantis*, *S*. *anginosus*, *S*. *phocae*, *S*. *agalactiae*, *S*. *sobrinus*, *S*. *pseudopneumoniae*, and *S*. *minor*. The relative abundance varied widely for Streptococcal species ranging from less than 0.0001% for *Streptococcus minor* to 3% for *S*. *infantis*. A total of 1496 unique OTUs identified at the genus level to be *Streptococcus* could not be identified at the species level.

### Alpha and beta diversity comparisons by betel nut history

The total number of bacterial sequences did not significantly differ between current chewers (mean 3,676; std. dev. 2,071); past chewers (mean 3,739, std. dev. 1,597); and never chewers (mean 3,068, std. dev. 1,438) (p = 0.38).

#### Alpha diversity

Within-sample (alpha) diversity did not differ between ever vs. never chewers, past vs. never chewers, current vs. past chewers, current vs. never chewers, and current vs. past/ever chewers. Alpha diversity was significantly lower among long-term (≥10 years) chewers compared to never chewers based on Shannon (p = 0.002), Chao1 (p = 0.011), Observed Species (mean number of taxa 37.9 and 48.0, respectively; p = 0.002), and PD Whole Tree (p = 0.028) indices (**Table 2**). Alpha diversity was also significantly lower in chewers with oral lesions vs. chewers and non-chewers with no lesions based on the PD whole tree index (p = 0.047). Alpha diversity was examined accounting for tobacco added to betel nut quid and cigarette smoking. Alpha diversity was significantly lower in current chewers who chewed without tobacco compared to current chewers using added tobacco based on Shannon (p = 0.041), Chao1 (p = 0.002), Observed Species (mean number of taxa 39.9 and 48.2, respectively; p = 0.007), and PD Whole Tree indices (p-0.001) Among non-smokers, alpha diversity was significantly lower in current betel nut users chewing without tobacco compared to never chewers based on Shannon (p = 0.012), Chao1 (p = 0.001), Observed Species (mean number of observed taxa 35.4 and 46.6, respectively; p = 0.007), and PD whole tree indices (p-0.008). To evaluate the influence of cigarette smoking separate from betel nut use, never betel nut chewers were compared by smoking status. Alpha diversity did not differ among smokers and non-smokers who had never chewed betel nut based on all indices.

**Table 2:** Comparison of Oral Bacteria Within-Sample (Alpha) Diversity by Betel Nut Chewing History^a^.

	Shannon				Chao1				Observed Species				PD Whole Tree			
Betel Nut Status	mean	std	t stat	p-value	mean	std	t stat	p-value	mean	std	t stat	p-value	mean	std	t stat	p-value
Long-term chewers (≥10 yr)	4.00	0.78	-3.173	**0.002**	129.08	47.35	-2.695	**0.011**	37.85	9.96	-3.454	**0.002**	3.80	1.17	-2.236	**0.028**
Never Chewers	4.67	0.74			169.40	65.37			47.98	11.83			4.52	1.18		
Current chewers with Oral Lesions	3.71	0.51	-1.029	0.322	123.14	50.99	0.626	0.544	32.60	7.08	-1.023	0.307	4.71	1.19	-1.925	**0.047**
Chewers & Non-Chewers without Oral Lesions	3.96	0.71			113.80	41.89			35.72	8.85			5.49	1.16		
Current chewers without added tobacco	4.17	0.78	-2.077	**0.041**	118.29	33.23	-3.888	**0.002**	39.95	9.16	-2.735	**0.007**	4.05	1.08	-3.461	**0.001**
Current chewers with added tobacco	4.63	0.93			176.27	74.94			48.19	13.60			5.29	1.63		
Nonsmoking current chewer without tobacco	3.81	0.80	-2.850	**0.012**	108.40	35.49	-3.242	**0.001**	35.37	9.14	-3.197	**0.007**	3.12	0.81	-2.987	**0.008**
Nonsmoking never chewer	4.58	0.75			152.69	45.14			46.57	11.51			4.12	1.16		
Never chewer current smoker	4.24	0.50	-0.121	0.908	141.33	63.24	0.646	0.524	40.52	7.82	-0.026	0.982	2.86	0.74	-0.382	0.698
Never chewer non-smoker	4.28	0.78			123.96	48.22			40.63	9.40			3.01	0.80		

^a^ 4 subjects with fewer than 100 sequences not included

#### Beta diversity

**Fig 3:**
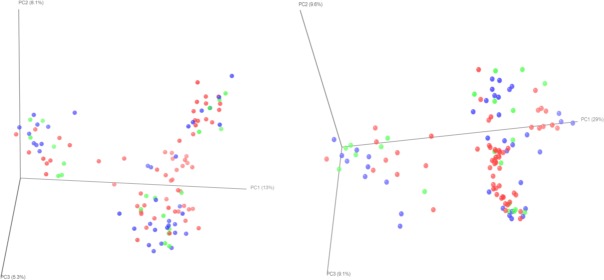
PCoA of UniFrac distance by betel nut chewing status: current (red); past (blue); never (green): **a.** unweighted: PERMANOVA (p = 0.016), ANOSIM (p = 0.026), MRPP (p = 0.027), PERMDISP (p = 0.256); **b.** weighted: PERMANOVA (p = 0.069), ANOSIM (p = 0.001), MRPP (p = 0.016), PERMDISP (p = 0.001).

Between-group (beta) diversity significantly differed by chewing status (**Table 3**). PCoA plots demonstrated clustering between current, past, and never chewers (**Fig 3A and 3B**). Unweighted unifrac distances significantly differed between current, past, and never chewers based on PERMANOVA (p = 0.016), ANOSIM (p = 0.026), and MRPP (p = 0.027). Weighted Unifrac distance was also significant between current, past, and never chewers based on ANOSIM (p = 0.001), MRPP (p = 0.016), and PERMDISP (p = 0.001). When current chewers were compared to past and never chewers combined, unweighted Unifrac distances significantly differed based on PERMANOVA (p = 0.003), ANOSIM (p = 0.006), and MRPP (p = 0.002); and weighted Unifrac distances were significantly different based on ANOSIM (p = 0.001), MRPP (p = 0.005), and PERMDISP (p = 0.001). Ever chewers (current and past chewers combined) and never chewers significantly differed based on unweighted and weighted ANOSIM (p = 0.032 and p = 0.012, respectively) and weighted PERMDISP (p = 0.037). Betel nut chewers with oral lesions and individuals without lesions differed in unweighted Unifrac distances based on PERMANOVA (p = 0.006), and ANOSIM (p = 0.001); and unweighted and weighted MRPP (p = 0.011 and p = 0.04, respectively). No differences in beta diversity was observed between long-term and never chewers.

**Table 3:** Comparison of beta diversity based on Unifrac distances by betel nut chewing status^a,b^.

				PERMANOVA		ANOSIM		MRPP		PERMDISP	
Comparison groups				Pseudo-F	p-value	R^c^	p-value	A^d^	p-value	F	p-value
Current chewers	Past chewers	Never chewers	Unweighted	1.388	**0.016**	0.052	**0.026**	0.0037	**0.027**	1.413	0.256
			Weighted	1.570	0.069	0.149	**0.001**	0.0090	**0.016**	9.096	**0.001**
Current chewers	Past/never chewers		Unweighted	2.017	**0.003**	0.044	**0.006**	0.0056	**0.002**	0.0001	0.99
			Weighted	1.570	0.074	0.149	**0.001**	0.0118	**0.005**	18.659	**0.001**
Ever chewers	Never chewers		Unweighted	1.062	0.338	0.089	**0.032**	0.0002	0.354	0.334	0.548
			Weighted	1.541	0.124	0.169	**0.012**	0.0020	0.190	4.517	**0.037**
Long-term chewers	Never chewers		Unweighted	1.281	0.091	0.000	0.432	0.0027	0.064	1.499	0.213
			Weighted	1.187	0.281	0.058	0.145	0.0031	0.186	1.767	0.178
Chewers with oral lesions	Chewers & non-chewers without oral lesions		Unweighted	1.893	**0.006**	0.346	**0.001**	0.0034	**0.011**	0.015	0.883
			Weighted	1.718	0.081	-0.011	0.507	0.0046	**0.040**	1.316	0.24
Betel nut with added tobacco	Betel nut without added tobacco		Unweighted	1.9186	**0.005**	0.0791	**0.005**	0.0076	**0.002**	0.2102	0.6483
			Weighted	2.9973	**0.005**	0.0706	**0.009**	0.0164	**0.008**	2.1916	0.1442
Nonsmoking current chewer without added tobacco	Nonsmoking never chewer		Unweighted	1.3704	0.06	0.0942	**0.042**	0.0052	0.091	0.4229	0.5197
			Weighted	1.2916	0.167	0.101	**0.035**	0.0093	0.065	0.5615	0.4587
Smoking non-chewers	Nonsmoking non-chewers		Unweighted	0.8559	0.741	-0.0072	0.496	-0.0043	0.75	0.8229	0.357
			Weighted	0.5361	0.867	-0.0669	0.647	-0.0158	0.939	0.0184	0.923

Beta diversity was examined accounting for tobacco exposure including both tobacco added to betel quid and cigarette smoking. Unifrac distances significantly differed between current chewers with and without added tobacco based on unweighted and weighted PERMANOVA (unweighted: p = 0.005; weighted: p = 0.005), unweighted and weighted ANOSIM (unweighted: p = 0.005; weighted: p = 0.009), and unweighted and weighted MRPP (unweighted: p = 0.002; weighted: p = 0.008). Beta diversity also significantly differed between nonsmoking current chewers without added tobacco compared to nonsmoking never chewers based on comparison of unweighted and unweighted ANOSIM (unweighted: p = 0.042; weighted: p = 0.035). When limited to those who had never chewed betel nut, beta diversity did not differ among smokers and non-smokers.

### Taxonomic comparisons by betel nut use

**Fig 4:**
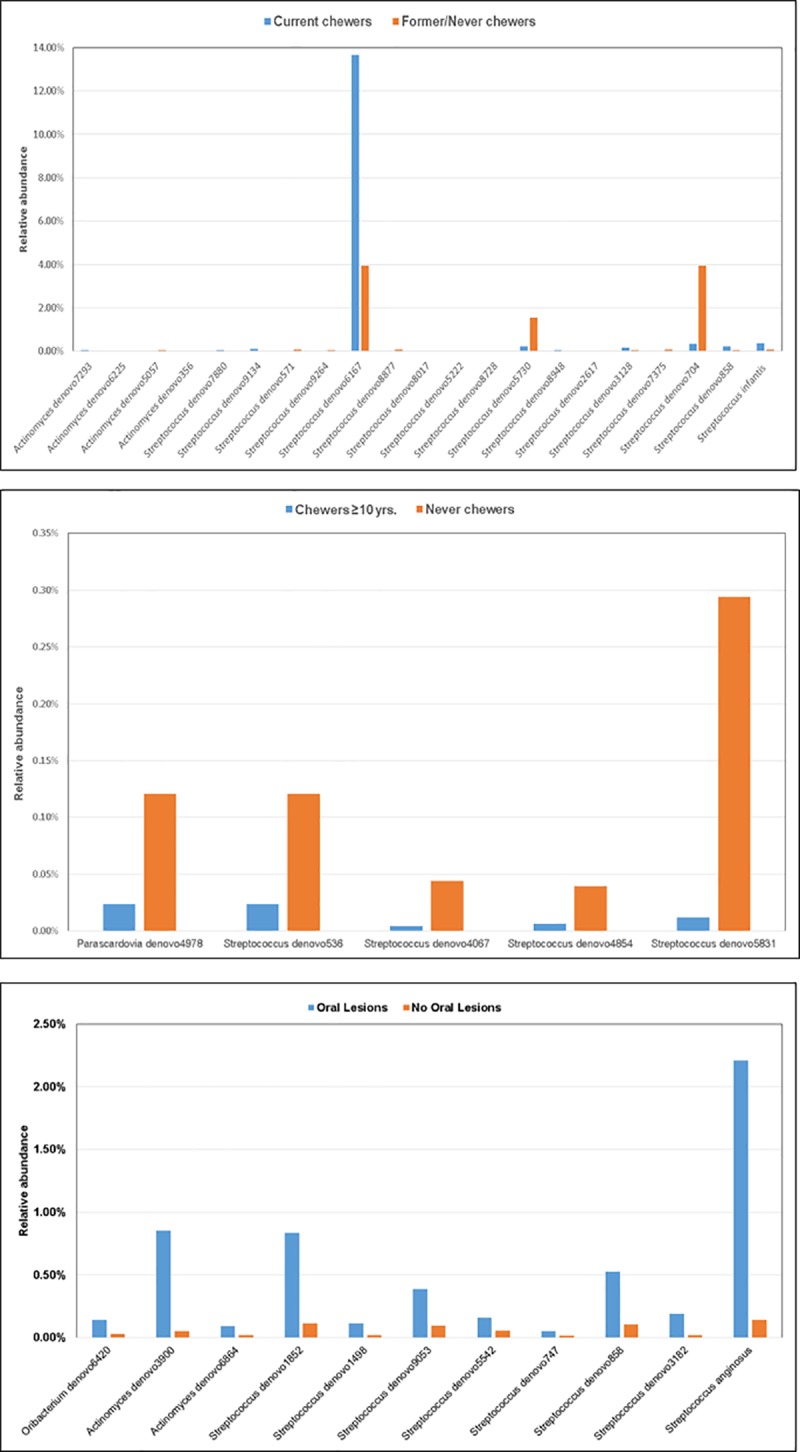
**a.** Relative abundance of oral bacteria taxa: current betel nut chewers vs. former/never chewers (FDR-corrected p<0.05). **b.** Relative abundance of oral bacteria taxa: long-term betel nut chewers (≥10 yrs.) vs. never chewers (FDR-corrected p<0.05). **c.** Relative abundance of oral bacteria taxa: betel nut chewers with oral lesions vs. chewers/non-chewers without oral lesions (FDR-corrected p<0.05).

The relative abundances of specific bacterial taxa were compared by betel nut use and oral lesion status. Significant differences (FDR-corrected p<0.05) in the relative abundance of prevalent genera and species were observed. Nine OTUs of the genus *Streptococcus* were detected at higher relative abundance in the oral cavity of current chewers compared to that of past/never chewers, including the highly abundant *Streptococcus* denovo6167 (14% in current chewers and 4% in past never chewers) (**Fig 4A**). Conversely, eight *Streptococcal* OTUs were each observed at lower relative abundance in current chewers including *Streptococcus* denovo704 (0.3% in current chewers and 4% in past/never chewers). The species, *Streptococcus infantis*, was nearly 4 times more abundant in current chewers compared to past/never chewers. Of the genus, Actinomyces, two OTUs were detected at higher relative abundance in current chewers compared to past/never chewers while two OTUs were at lower relative abundance. Compared to never chewers, long-term chewers (≥10 years) had reduced levels of one OTU of the genus *Parascardovia* and four *Streptococcal* OTUs (**Fig 4B**). Compared to chewers and non-chewers without oral lesions, betel nut chewers who presented with oral lesions had higher levels of *Oribacterium* (1 OTU), *Actinomyces* (2 OTUs), and *Streptococcus* (8 OTUs) compared to individuals without lesions (**Fig 4C**). The species, *Streptococcus anginosus*, was at 16-fold higher relative abundance in betel nut chewers with oral lesions. Comparison of the relative abundance of OTUs showed no significant differences in specific bacterial taxa in chewers with and without tobacco based on FDR-corrected p-values.

### Comparison of bacterial diversity and taxonomy by other factors

Alpha and beta diversity indices did not vary by gender, BMI, excessive alcohol consumption, cigarette smoking, diabetes history, and oral HPV DNA (p≥0.05 for all indices). Beta diversity significantly differed by age and antibiotic use. Beta diversity among individuals aged 18–39 years and 40 years and older differed based on Unifrac distances by PERMANOVA (unweighted: p = 0.006; weighted: p = 0.019), ANOSIM (unweighted: p = 0.011; weighted: p = 0.032), MRPP (unweighted: p = 0.003; weighted p = 0.033). Beta diversity among individuals using and not using antibiotics within the prior 6 months differed based on ANOSIM (unweighted: p = 0.043; weighted: p = 0.019), MRPP (weighted p = 0.02), and PERMDISP (unweighted p = 0.04284).

Given the differences in beta diversity by age and antibiotic use, these and other factors were further examined stratified by chewing status. When separately examined in current chewers and in never chewers, neither alpha diversity nor beta diversity indices differed for any factor with the exception of alcohol consumption. Beta diversity significantly differed by alcohol use only among current chewers. Among current chewers, unweighted Unifrac distances differed between drinkers and non-drinkers by PERMANOVA (unweighted p = 0.04) and ANOSIM (unweighted p = 0.045). No difference was observed by alcohol consumption among non-chewers.

There were no differences in the relative abundance of specific bacterial taxa by age, gender, BMI, excessive alcohol consumption, cigarette smoking, and oral HPV DNA. The genus, *Streptococcus*, was observed at higher relative abundance in antibiotic users compared to non-users (FDR-corrected p<0.05). The relative abundances of the genera, *Parascardovia* and *Capnocytophaga*, were higher than in diabetics compared to non-diabetics (FDR-corrected p<0.05).

### Predictors of oral premalignant lesions

The relationship of oral premalignant lesions with chewing status and other factors was further examined. *Streptococcus infantis* and *Streptococcus anginosus*, were specifically examined as they were shown to be significantly elevated in current chewers and chewers with oral lesions, respectively. Nine of 10 (90%) individuals with oral lesions were current betel nut users compared to 55 of 112 (49%) of those without lesions (p = 0.013) (unadjusted OR 9.32, 95% CI 1.14–76.05) (**Table 4**). HPV was detected in 4 of the 10 (40%) individuals with oral premalignant lesions (including the 1 case of OSF) and 13 of 112 (12%) individuals without lesions (p = 0.013) (unadjusted OR 5.08, 95% CI 1.26–20.40). All 10 individuals with oral lesions were cigarette smokers compared to 68.8% (77/112) of those without oral lesions (p = 0.036), all were positive for *S*. *anginosus* compared to 87.5% (98/112) of those without lesions (p = 0.235), and 80% (8/10) of individuals with oral lesions were positive for *S*. *infantis* compared to 93.8% (105/112) without lesions (p = 0.111). The presence of oral lesions did not differ by age, gender, antibiotic use, alcohol consumption, diabetes history, or BMI (data not shown). In multivariate models which included oral HPV and levels of *S*. *anginosus and S*. *infantis* as covariates, current betel nut chewing remained the only predictor of oral premalignant lesions (adjusted OR 10.39, 95% CI 1.08–100.28).

**Table 4:** Relationship of oral premalignant lesions with betel nut chewing and other factors.

				Unadjusted		Adjusted^b^	
	Oral Lesions (n = 10)	No Oral Lesions (n = 112)	Chi square p-value	Odds Ratio	95% Confidence Interval	Odds Ratio	95% Confidence Interval
***Betel nut chewing***							
Current chewer	9	55	**0.013**	**9.32**	**1.14–76.05**	**10.39**	**1.08–100.28**
Past/never chewer	1	57					
***Cigarette smoking***							
Smoker	10	77	**0.036**	N/A^a^		N/A^a^	
Non-smoker	0	35					
***Streptococcus infantis***							
Positive	8	105	0.111	0.96	0.91–1.02	0.93	0.85–1.02
Negative	2	7					
***Streptococcus anginosus***	** **	** **					
Positive	10	98	0.235	N/A^a^		N/A^a^	
Negative	0	14					
***Oral HPV DNA***	** **	** **	** **	** **	** **	** **	** **
Positive	4	13	**0.013**	**5.08**	**1.26–20.40**	6.84	0.99–47.31
Negative	6	99					

^a^ Odds ratios could not be estimated due to null cell values

^b^ Adjusted for current betel nut chewing (yes/no), smoking (yes/no), oral HPV DNA (positive/negative), *Streptococcus infantis (relative abundance)*, *Streptococcus anginosus (relative abundance)*

## Discussion

Our study provides evidence that betel nut chewing alters the oral bacterial microbiome. The oral microbiome of betel nut chewers demonstrated reduced richness and evenness and shifts changes in the relative abundance of common bacteria. Microbiome alternations were most pronounced in current chewers, particularly those using betel nut for 10 years or longer. Although some microbial differences were seen in formal betel nut chewers, most of the differences were observed in current chewers, which may indicate that disruption of the oral microbiome induced by betel nut chewing is reversible.

Prolonged use of betel nut may disrupt the bacterial composition of the oral including reduced levels of commensal bacteria critical to maintaining homeostasis. The reduction in the predominant bacterial species may be accompanied by increases in levels of other, less abundant bacteria. Indeed, we observed that betel nut chewers had significant elevation or reduction in the relative quantities of common oral bacteria including *Streptococcus* which, consistent with other studies [14, 15], was the predominant genus of the oral microbiome. *Streptococcus infantis*, was observed at levels 4-fold higher in current chewers compared to past/never chewers. Specific genus-level *streptococcal* OTUs, each presumably representing other distinct *Streptococcal* species, were either significantly more or less abundant in current betel nut users compared to past or never users. Similarly, genus-level *Actinomyces* OTUs were significantly elevated or reduced in current chewers.

There is *in vitro* evidence that betel nut may have anti-bacterial properties which may explain the reduction in some bacteria taxa. Prolonged exposure to the aqueous extracts of betel nut and, in particular, tannic acid, have been shown to suppress the growth of common *Streptococcal* species, cultured from saliva.[49] Aqueous betel nut extracts also inhibit the growth of *Streptococcus intermedius*, *S*. *anguinis*, *and S*. *mutans* from saliva and supragingival plaque samples.[50]

It is unclear changes in the oral microbiome caused by betel nut chewing may contribute to oral cancer development. Notably, alterations in the oral microbiome were observed among chewers presenting with oral premalignant lesions, including leukoplakia and submucous fibrosis. In addition to differences in both alpha and beta diversity indices, betel nut chewers with oral lesions exhibited elevated levels of a number of specific Streptococcal OTUs, as well as OTUs of the *Oribacterium* and *Actinomyces*. Notably, *Streptococcus anginosus* levels were 16-fold higher in betel nut chewers with oral lesions compared to individuals with no lesions. *S*. *anginosus* has been previously detected in tumor tissue of oral squamous cell carcinoma patients [21, 51–53] as well as non-tumorous tissue contiguous to tumor and in dental plaque from oral SCC patients.[28, 53] Notably, *S*. *anginosus*, an anaerobic bacteria, has been found to induce the synthesis of NO and inflammatory cytokines in murine models,[54] suggesting potential mechanisms of carcinogenesis. A number of other Streptococcal species, *including S*. *salivarius*, *S*. *gordonii*, *S*. *parasanguinis*, have been observed in greater abundance in oral squamous cell carcinoma compared to non-tumor tissue.[28]

Whether changes in the oral microbiome in betel nut chewers influences oral carcinogenesis is entirely speculative. It is possible that reduction in commensal species facilitates the emergence of harmful bacteria although no pathogenic species were significantly elevated in our study population. Alternatively, disruption in the normal oral microflora may impede its ability to counter betel nut-induced inflammation of the oral mucosa resulting in increased susceptibility to malignant transformation.

Consistent with the multifactorial etiology of oral cancers, current betel nut chewing, cigarette smoking, and oral HPV were each significantly more prevalent in those presenting with oral premalignant lesions. However, when accounting for smoking, HPV, and levels of *S*. *anginous* and *S*. *infantis*, only current betel nut use remained as a significant predictor of oral lesions. This affirms betel nut chewing as an independent risk factor for oral cancer. Moreover, betel nut-induced changes in the oral microbiome may unrelated to oral carcinogenesis.

Tobacco use, including cigarette smoking and tobacco chewing, is the primary cause of oral cancer worldwide.[2] Cigarette smoking has also been associated with changes in the oral microbiome including significant increases and decreases in the abundance of common taxa.[55] We observed that alpha and beta bacterial diversity significantly differed between current betel nut users who chewed with and without added tobacco. In particular, within-sample taxa diversity was higher in chewers who added tobacco. This is consistent with a previous study which found that the bacterial composition of the oropharynx is significantly more diverse in smokers than in nonsmokers.[56] Betel nut use and smoking may have different influences on the oral cavity—respectively decreasing and increasing taxa richness and evenness. However, among participants with no history of betel nut use, microbial diversity did not differ between smokers and non-smokers. Importantly, the altered oral microbiome of betel nut users appears to be distinct from the influences of tobacco. Within-sample diversity was significantly lower in nonsmoking current betel nut users who chewed without added tobacco compared to nonsmoking never chewers, indicating that the changes in microbial diversity observed in betel nut chewers were due to betel nut use alone rather than cigarette smoking.

Excessive alcohol consumption may explain some of the oral microbial differences observed with betel nut chewing. Based on unweighted indices, beta diversity significantly differed by excessive alcohol use among those who were current chewers but not among never chewers. This indicates that chewing of betel nut, when combined with high levels of alcohol, may alter the oral microbiome. Notably, in the presence of ethanol, certain bacterial species are able to produce high levels of acetaldehyde, an oral carcinogen.[57] As unweighted UniFrac distance is generally able to detect changes in rare rather than abundant taxa [43], it is likely that any combined influence of betel nut chewing and alcohol was limited to bacterial taxa occurring less frequently in the oral cavity

We were able to evaluate the potential role of other factors that may have influenced the relationship of betel nut chewing and the oral microbiome. Overweight and obesity was significantly more prevalent among current chewers compared to past and never chewers. This is consistent with evidence linking betel nut use with obesity.[58, 59] Nonetheless, alpha and beta diversity and the abundance of specific bacterial taxa did not significantly vary by BMI, indicating that the influence of betel nut use on the oral microbiome was independent of body size.

Beta diversity significantly differed by age and antibiotic use within the past 6 months. The relative abundance of *Streptococcus* was significantly higher in antibiotic users compared to non-users perhaps reflecting the increased establishment of non-pathogenic *Streptococcal* species following the reduction in pathogenic oral bacterial with antibiotic treatment. We also observed differences by diabetes history with higher abundance of 2 genera, including *Capnocytophaga*. High levels of *Capnocytophaga* in diabetes mellitus have been previously reported.[60, 61] Nonetheless, it is unlikely that the observed differences in the microbiome of betel nut users was influenced by age, antibiotic use, or diabetes as betel nut use did not vary by these factors.

Compared to other sites of human body, the oral microbiome has a high degree of variability of taxa composition within individuals while having less variability across individuals.[62] Notably, we observed differences in bacterial diversity both within and between individuals. The oral microbiome varies between saliva and the oral cavity as well as between subsites of the oral cavity, such as the buccal mucosa and supragingival and subgingival plaque. [63] A limitation of the study was the lack of separate evaluation for saliva and oral swabbings, as well as separate evaluation of different subsites of the oral cavity.

The inability to identify most taxa at the species level is a major limitation of our investigation and most other next generation sequencing-based studies of the microbiome. The importance of species-level identification was underscored by our findings that some OTUs of Streptococcus, which comprised half of genus-level OTUs, were significantly elevated while others were significantly reduced in betel nut chewers.

To date, the role of betel nut chewing in oral carcinogenesis is poorly understood. Our study provides evidence that betel nut chewing alters the oral bacterial microbiome including that of chewers who develop oral premalignant lesions. Nonetheless, whether microbial changes are involved in betel nut-induced oral carcinogenesis is only speculative. Further avenues of research are needed to address the clinical significance of an altered microbiome including any potential role in oral cancer development. This includes metagenomic and metabolomic studies to identify bacterial genes and metabolites that may influence oral carcinogenesis.

## Supporting information

S1 File Survey Instrument. (PDF)
