# Supplementary material for: Betel nut chewing, oral premalignant lesions, and the oral microbiome
Source: PLoS One. 2017 Feb 22;12(2):e0172196. doi: 10.1371/journal.pone.0172196 (PMC5321455; doi:10.1371/journal.pone.0172196)
Supplement: S1 File — (PDF) [file pone.0172196.s001.pdf]

DEMOGRAPHIC INFORMATION

ID #__________________ Date ____________________ Interviewer Initials_____________

Date of Birth:_________________ Age:_________________

Gender: ☐ Male ☐ Female Island/Country of birth: ________________

How many total years of your life have you lived on Guam or Saipan? _____________Years

Identify your ethnic background by indicating the percentage in the space provided below. The total overall should equal 100% when complete.

	%		%
Chamorro (Guam)		Pohnpeian	
Chamorro (CNMI)		Pohnpeian (outer island)	
Carolinian (Caroline Islands)		Yapese	
Chuukese		Yapese (outer island)	
Chuukese (outer island)		Filipino	
Kosraean		Other (specifiy):	
Kosraean (outer island)			
Marshallese			
Palauan			

TOTAL % _________

Which **ONE** ethnic group that you most identify with?_______________________

What is you annual household income?

☐ Less than $10,000
☐ Between $10,000 to $20,000
☐ Between $20,000 to $30,000
☐ Between $30,000 to $40,000
☐ Between $40,000 to $50,000
☐ Between $60,000 to $70,000
☐ $70,000 or more

What is the highest education level you have completed?

☐ Elementary school (K-5)
☐ Middle School (6-8)
☐ High School (9-12)
☐ Some college
☐ 4-year College Degree (bachelor's degree)
☐ Some Graduate School
☐ Graduate Degree (MS, PhD, MD, JD)

Marital Status:

☐ Single
☐ Married
☐ Divorced
☐ Separated
☐ Widowed

PERSONAL MEDICAL HISTORY

ID #__________________ Date ____________________ Interviewer Initials _________________

Has a medical doctor ever told you that you had/have… (Please circle answer)	If yes, what kind? (Please circle)	If yes, how old were you when you were first told you had…	Do/Did either of you parents have… (Please circle answer)
1. Diabetes? Yes No	Type 1 Type 2 Gestational	_________________ years	Yes No<br>If yes: Mom ____ Dad____
2. Heart attack?<br>Yes No		_________________ years	Yes No<br>If yes: Mom ____ Dad____
3. Stroke?<br>Yes No		_________________ years	Yes No<br>If yes: Mom ____ Dad____
4. Hypertension? (High Blood Pressure)<br>Yes No		_________________ years	Yes No<br>If yes: Mom ____ Dad____

ORAL HEALTH BEHAVIOR QUESTIONNAIRE

ID #__________________ Date ____________________ Interviewer Initials ____________

BETEL NUT

1. Have you ever chewed betel nut in your life?

☐

1	Yes	
2	No	**[Go to 17]**
7	Don't know/Not sure	**[Go to 17]**
9	Refused	**[Go to 17]**

2. Do you now chew betel nut?

☐

1	Yes	
2	No	
7	Don't know/Not sure	**[Go to 17]**
9	Refused	**[Go to 17]**

3. How often (do/did) you chew betel nut?

☐

1	Daily	
2	Weekly	
3	Monthly	
7	Don't know/Not sure	**[Go to 5]**
9	Refused	**[Go to 5]**

4. How many times per day, week, or month (do/did) you chew betel nut?

☐

______ times
7 Don't know/Not sure
9 Refused

5. (Do/Did) you include lime when chewing betel nut?

☐

1	Yes	
2	No	**[Go to 7]**
7	Don't know/Not sure	**[Go to 7]**
9	Refused	**[Go to 7]**

6. How (do/did) you obtain your lime?

☐

1 Homemade (Complete Lime Recipe Form)
2 Store (Identify store: ____________________)

3 Family/friend
7 Don't know/Not sure
9 Refused

7. (Do/Did) you include tobacco when chewing betel nut? (See Tobacco Guide for type.)
☐
1 Yes (Identify type: ___________________)
2 No
7 Don't know/Not sure
9 Refused

8. (Do/Did) you include *pupulu* or pepper leaf when chewing betel nut?
☐
1 Yes
2 No
7 Don't know/Not sure
9 Refused

9. What variety of betel nut (do/did) you most often chew?
☐
1 Red
2 White
7 Don't know/Not sure
9 Refused

10. What stage of betel nut (do/did) you most often chew?
☐
1 Young (unripe)
2 Mature (ripe)
7 Don't know/Not sure
9 Refused

11. (Do/Did) you add alcohol to any of the components of your chew (nut, leaf, lime or tobacco)?
☐
1 Yes
2 No
7 Don't know/Not sure
9 Refused

12. (When you [chew/chewed] betel nut) How long do you keep the chew (nut) in your mouth?
☐

______ minutes
7 Don't know/Not sure
9 Refused

13. When you [sleep/slept] at night, (do/did) you keep the chew (nut) in your mouth?
☐

1 Yes
2 No
7 Don't know/Not sure
9 Refused

14. (Do/Did) you ever ingest (swallow) your chew?
☐

1 Yes
2 No **[Go to 16]**
7 Don't know/Not sure **[Go to 16]**
9 Refused **[Go to 16]**

15. How often (do/did) you ingest (swallow) your chew?
☐

1 Sometimes
2 Most of the times
3 All the time
7 Don't know/Not sure
9 Refused

16. How long have you been chewing betel nut?
☐

______ years
7 Don't know/Not sure
9 Refused

SMOKELESS TOBACCO

17. Aside from adding tobacco to a betel quid, how often do you use smokeless tobacco?
☐

1 Everyday
2 Somedays
3 Not at all
7 Don't know/Not sure
9 Refused

CIGARETTE SMOKING

18. Do you smoke cigarettes at least once per day?
☐

1 Yes
2 No **[Go to 20]**

7 Don't know/Not sure **[Go to 20]**
9 Refused **[Go to 20]**

19. How many sticks do you smoke daily?
☐
______ sticks
7 Don't know/Not sure
9 Refused

ALCOHOL

20. Did you drink alcohol within the past 30 days?
☐
1 Yes
2 No **[Go to 23]**
7 Don't know/Not sure **[Go to 23]**
9 Refused **[Go to 23]**

21. During the past 30 days, on the days when you drank, about how many drinks did you drink? [Refer to Alcohol Guide for types and size.]
☐
______ TOTAL UNITS

______ cans of beer

______ bottles of beer

______ cups tuba/kava

______ glasses of mixed drink

______ glasses of wine

______ shots of liquor

7 Don't know/Not sure
9 Refused

22. During the past 30 days, what is the largest number of drinks you had on any occasion?
☐
______ TOTAL UNITS

______ cans of beer

______ bottles of beer

______ cups tuba/kava

______ glasses of mixed drink

______ glasses of wine

______ shots of liquor

7 Don't know/Not sure
9 Refused

ORAL CARE

23. Have you ever been told by a dentist or doctor that you have cancer of the mouth?
☐
1 Yes
2 No
7 Don't know/Not sure
9 Refused

24. How often do you brush your teeth?
☐
1 More than once per day
2 Once per day
3 Once per week
4 Once per year
5 Never
7 Don't know/Not sure
9 Refused

25. Do you use the husk of the betel nut for cleaning your teeth?
☐
1 Yes
2 No
7 Don't know/Not sure
9 Refused

26. Do you visit your dentist at least once a year?
☐
1 Yes
2 No
7 Don't know/Not sure **[End]**
9 Refused **[End]**

Medication and Sexual History

ID #_________________ Date ___________________ Interviewer Initials____________

Interviewer will start by stating:

Sexual history influences HPV status, and thus is a risk factor for oral cancer. The questions below will help researchers better understand this risk in betel nut chewers.

1. Within the past 6 months, have you taken antibiotics? ___YES ___NO

2. Within the past 6 months, have you taken any of the following medications at least 2 times per week for 1 month or longer? (check all that apply)
__ aspirin (e.g., Bayer, Bufferin, Excedrin)
__ ibuprofen (e.g., Advil, Motrin, Nuprin)
__ Naproxen (e.g., Aleve)
__ prescription pain medication
__ Other NSAIDS
__ acetaminophen (e.g.,Tylenol)

3. Have you ever been diagnosed with a sexually transmitted infection?
__YES __NO

If yes, please describe:____________________

4. (Females only) Have you ever had an abnormal Pap smear?
__YES __NO

5. Have you ever had sexual contact with a male? YES NO (if NO, skip to question #10)

6. How many different males have you had sexual contact with over your lifetime? ______

7. What type of sexual contact have you had with a male? (check all that apply)
___ Vaginal intercourse
___Anal intercourse
___Oral (received oral sex)
___Oral (performed oral sex)
___Other (describe)_______

8. Have you had sexual contact with a male within the past 3 months? YES NO (if NO, skip to question #10)

9. What type of sexual contact have you had with a male within the past 3 months? (check all that apply)

___ Vaginal intercourse
___Anal intercourse
___Oral (received oral sex)
___Oral (performed oral sex)
___Other (describe)_______

10. Have you ever had sexual contact with a female? YES   NO (if NO, skip to END)

11. What type of sexual contact have you had with a female? (check all that apply)

___ Vaginal intercourse
___Anal intercourse
___Oral (received oral sex)
___Oral (performed oral sex)
___Other (describe)_______

12. Have you had sexual contact with a female within the past 3 months?   YES   NO (if NO, skip to END)

13. What type of sexual contact have you had with a female within the past 3 months? (check all that apply)

___ Vaginal intercourse
___Anal intercourse
___Oral (received oral sex)
___Oral (performed oral sex)
___Other (describe)_______

Physical Assessment

ID # ________________ Date ______________ Screener Initials ______________

Before you begin, be sure that the individual has not eaten or drank any beverage for at least an hour. Also, ensure that the individual has rid him/herself of wallets, watches, jewelry, or any other material that may skew results.

	TRIAL 1	TRIAL 2	TRIAL 3	AVERAGE
**WEIGHT (lbs.)**				
**HEIGHT (in.)**				
**BLOOD PRESSURE (mmHg)***	____/____	____/____	____/____	____/____
*** Normal Blood Pressure would be about 120/70. *High* Blood Pressure is when the diastolic number is above 80mmHg. *Low* Blood Pressure is when the diastolic number is below 60mmHg.**				

BODY MASS INDEX (BMI):

$$\text{BMI} = 703 \times \frac{\text{Weight (lbs.)}}{\text{Height}^2\text{ (in.)}}$$

BMI= 703 x (________) =
( )

**Under Weight**	**<18.5**
**Normal Weight**	**18.5-24.9**
**Over Weight**	**25-29.9**
**Obesity**	**30<**

BLOOD PRESSURE:

	Systolic	Diastolic
**Low**		**<60 mmHg**
**Normal**	**120 mmHg**	**70 mmHg**
**High**		**>80 mmHg**
